# Fast and Reliable Differentiation of Eight *Trichinella* Species Using a High Resolution Melting Assay

**DOI:** 10.1038/s41598-017-16329-x

**Published:** 2017-11-24

**Authors:** Nikol Reslová, Lucie Škorpíková, Michal Slaný, Edoardo Pozio, Martin Kašný

**Affiliations:** 1 Veterinary Research Institute, Department of Food and Feed Safety, Hudcova 296/70, 621 00 Brno, Czech Republic; 2 Faculty of Science, Department of Botany and Zoology, Masaryk University, Kamenice 5, 625 00 Brno, Czech Republic; 3 European Union Reference Laboratory for Parasites, Istituto Superiore di Sanita, viale Regina Elena 299, 00161 Rome, Italy

## Abstract

High resolution melting analysis (HRMA) is a single-tube method, which can be carried out rapidly as an additional step following real-time quantitative PCR (qPCR). The method enables the differentiation of genetic variation (down to single nucleotide polymorphisms) in amplified DNA fragments without sequencing. HRMA has previously been adopted to determine variability in the amplified genes of a number of organisms. However, only one work to date has focused on pathogenic parasites–nematodes from the genus *Trichinella*. In this study, we employed a qPCR-HRMA assay specifically targeting two sequential gene fragments–*cytochrome c oxidase subunit I* (*COI*) and *expansion segment V* (*ESV*), in order to differentiate 37 single L1 muscle larvae samples of eight *Trichinella* species. We show that qPCR-HRMA based on the mitochondrial *COI* gene allows differentiation between the sequences of PCR products of the same length. This simple, rapid and reliable method can be used to identify at the species level single larvae of eight *Trichinella* taxa.

## Introduction

Zoonotic cosmopolitan nematodes of the genus *Trichinella* are causative agents of human trichinellosis, a serious human disease^1^, which has been documented in 55 countries around the world^2^. A broad range of carnivore and omnivore (mammals, birds, and reptiles) animals have also been reported to be hosts of these parasites, including the economically important domestic pig^1^. Among the various *Trichinella* hosts, only humans develop a serious clinical infection, which can lead to death^3^; therefore, trichinellosis is strictly monitored in the context of animal trade and food safety^4^. In Europe according to the Commission Regulation No. 2015/1375, all *Trichinella* susceptible animals intended for human consumption shall be tested for *Trichinella* spp. larvae and isolated larvae shall be identified at the species level.

Based on genetic, zoogeographical and epidemiological characters, 12 taxa are recognised in the genus *Trichinella*, which is separated in two clades one that encompasses species that encapsulate in host muscle tissues following muscle cell reprogramming, and a second that does not encapsulate^5,6^. The encapsulated clade contains six species (*T*. *spiralis*, *T*. *nativa*, *T*. *britovi*, *T*. *murrelli*, *T*. *nelsoni*, and *T*.*patagoniensis*) and three genotypes (*Trichinella* T6, T8, and T9). Infectious larvae (L1) of these species can develop only in mammals, where they induce the transformation of muscle cells in a typical nurse cell surrounded by a collagenous layer^7^. The three representatives of the non-encapsulated clade are known to infect not only mammals, but also birds (*T*. *pseudospiralis*) and reptiles (*T*. *papuae* and *T*. *zimbabwensis*). The L1 muscle larvae of these species are surrounded only by a thin collagenous layer^8^. Despite this differentiation based on whether the species form capsules or not, there are no unambiguous morphological features useful for species differentiation.

The identification of *Trichinella* species is, to a large extent, based on multiplex PCR analyses of ribosomal DNA (rDNA) fragments and on variability in their lengths, which manifests as a unique electrophoretic DNA banding pattern^8–12^. Currently, the *expansion segment V* (*ESV*) of the large subunit of rDNA (LSU rDNA) and repeat sequences of the *internal transcribed spacers* 1 and 2 (*ITS1*, *ITS2*) are mostly used as standard molecular sequence targets^13^. By this approach, it is possible to differentiate all currently defined species, including three genotypes of *T*. *pseudospiralis* (from the Australian, Nearctic, and Palearctic regions) and the T6 genotype. Additionally, the PCR-restriction fragment length polymorphism (RFLP) analysis of the gene encoding a 43 kDa excretory/secretory antigen digested with the endonuclease SspI^14^ or the mitochondrial (mt) partial *cytochrome c oxidase subunit I* (*COI*) gene^15^ digested with MseI enable differentiation of the T9 genotype. If the same product is digested with AluI the T8 genotype can also be recognized^15^.

High resolution melting analysis (HRMA) was originally intended for genotyping, mutation scanning, and sequence matching, however, it might also be suitable for species identification, since the melting profile of a PCR product and the shape of the HRM species-specific matrix curves depend on GC content, length, and nucleotide sequence^16^. In last years, this approach became frequently used for a various pathogens identification, including parasites and microorganisms. It was successfully applied, e.g. in determination of haplotypes of giant liver fluke *Fasciolides magna* (7 haplotypes)^17^, identification of medically important *Candida* spp. (21 species)^18^ or bacteria (37 species)^19^. A qPCR assay in combination with HRMA has been developed for detection of polymorphisms in *Trichinella ESV* ^20^, resulting in the genotyping of four species–*T*. *spiralis*, *T*. *nativa*, *T*. *britovi*, and *T*. *pseudospiralis*. However, it was found that variations between the repeat sequences derived from a single isolate (intra-isolate) were higher than between isolates (inter-isolate) of a given parasite species, which led to the generation of non-overlapping HRM species-specific matrix curves.

The aim of our study was to develop a qPCR-HRMA method based on polymorphisms of the mt *COI* gene, which shows divergence even among closely related species^21^, but exhibits conservation within a particular species^22^. Such approach could bring many advantages, in comparison to currently available methods (such as multiplex PCR or RFLP analysis), in a form of a very easy fashion and requirement of minimal amount of sample – 1 larva. The single larva qPCR-HRMA enables reliable species determination without the risk of amplification bias and the need of any additional confirmations, such as sequencing. We analyzed the genomic DNA (gDNA) isolated from single muscle larva of eight *Trichinella* species (*T*. *spiralis*, *T*. *nativa*, *T*. *britovi*, *T*. *pseudospiralis*, *T*. *nelsoni*, *T*. *murrelli*, *T papuae*, and *T*. *zimbabwensis*). Additionally, primers used for amplification of the *Trichinella ESV* region used in the previous HRMA study of Masny *et al*.^20^ were tested. This dual approach enabled us to better evaluate the potential of HRMA for *Trichinella* species determination.

## Materials and Methods

### Trichinella isolates

Muscle larvae (ML) of eight *Trichinella* species (Table 1) were provided by the International *Trichinella* Reference Center, Rome, Italy (https://www.iss.it/site/Trichinella/scripts/dedb.asp?lang=2). Larvae were preserved in 96% ethanol and stored at −20 °C until use. ML collected from naturally infected wild boar hunted in Poland were kindly provided by Dr. Mirek Rozycki (National Veterinary Research Institute in Pulawy/PIWet, Poland) and included in the study as blind samples.

**Table 1:** The origins of the nine muscle larvae isolates of *Trichinella* species.

Isolate code	Species	Host origin	Geographical origin	No. of ML analyzed
ISS3	*T*. *spiralis*	Domestic pig (*Sus scrofa domesticus*)	Poland (Warsaw)	4
ISS10	*T*. *nativa*	Polar bear (*Ursus maritimus*)	Norway (Svalbard islands)	4
ISS2	*T*. *britovi*	Red fox (*Vulpes vulpes*)	Italy (Sardinara)	4
ISS13	*T*. *pseudospiralis*	Raccoon (*Procyon lotor*)	Russia (Caucasus)	3
ISS588	*T*. *pseudospiralis*	Brown rat (*Rattus norvegicus*)	Russia (Kamchatka)	3
ISS37	*T*. *nelsoni*	Warthog (*Phacochoerus aethiopicus*)	Tanzania UR	4
ISS35	*T*. *murrelli*	Black bear (*Ursus americanus*)	USA (Pennsylvania)	4
ISS572	*T*. *papuae*	Wild pig (*Suis sp*.)	Papua New Guinea (Bula Plain)	4
ISS1029	*T*. *zimbabwensis*	Nile crocodile (*Crocodylus niloticus*)	Zimbabwe (Victoria falls)	3
Sample 1, 2		Wild boar (*Sus scrofa*)	Poland (Lublin)	2
Sample 3, 4		Wild boar (*Sus scrofa*)	Poland (Kuyavian-Pomeranian)	2

Two more isolates represented by samples 1–4 originated from natural infections and served as blind samples for study verification. ML – single muscle larva.

### DNA extraction

To properly reflect sample diversity and to balance isolate numbers, gDNA was extracted from at least three single ML (Table 1) from each reference species, in order to evaluate the genetic variability (later expressed as confidence intervals). DNA from two ML of each of two different host individuals (isolates), corresponding to blind samples, were extracted. In total, 37 ML were prepared for the present study.

Single individual larvae were collected from the larva pool under a dissection microscope and incubated at 55 °C overnight in 100 µl extraction buffer (100 mM Tris–HCl, 10 mM EDTA, 100 mM NaCl, 1% SDS, 1.5 mM dithiothreitol) containing 0.06 mg proteinase K^23^. To precipitate proteins after incubation, 3 M sodium acetate (1/3 of the lysate volume) and 5 µl oyster glycogen (20 mg/ml stock; SERVA) were added to the lysate and vortexed. DNA-containing supernatant was precipitated using a double volume of ice-cold 99.5% isopropanol. To increase the yield of nucleic acid, the samples were incubated at −70 °C for 30 min. After centrifugation, the DNA pellet was washed using 200 µl of 70% ethanol. Finally, the DNA pellet was dried in a heater and dissolved in 25 µl of molecular grade H_2_O. Samples were stored at −20 °C pending further processing.

By NanoDrop 2000c Spectrophotometer (Thermo Scientific) measurement was controlled the purity of isolated nucleic acid and the larval range of gDNA concentration was determined from 3 to 12 ng/μl.

### qPCR and HRMA

The conditions of the PCR reaction were adjusted for successful amplification of target gene fragments of the eight *Trichinella* species. The uniTrich1bis and Tsr1 PCR primers (Table 2) used for the *ESV* amplification were adopted from Masny *et al*.^20^; primer ESV_Rev1 and the degenerate primers for the *COI* gene FW1, FW2, and uniREV, were designed for this study according to the GenBank reference sequences: *T*. *spiralis* (AF293969.1), *T*. *nativa* (NC_025752.1), *T*. *britovi* (NC_025750.1), *T*. *pseudospiralis* (NC_025749.1), *T*. *nelsoni* (NC_025753.1). *T*. *murrelli* (NC_025751.1), *T*. *papuae* (NC_025754.1) and *T*. *zimbabwensis* (NC_025755.1).

**Table 2:** PCR primers for amplification of target genes in eight *Trichinella* species.

Target gene	Primer name	Sequence 5′->3′	Amplicon size
*COI*	FW1	TCAGGAGGAGG**R**GACCCCAT	531 bp
	FW2	tgtgtgAGATGA**Y**TAGCTACA**Y**TATA**Y**GG	240 bp
	uniREV	TCATGGTGTTCATA**R**TGTTACTGCGATT	
*ESV*	uniTrich1bis^a^	CTAAGAAAACGGCGAAAGC	
	ESV_Rev1	TCGGCGTTTTATGGATACC	313–468 bp
	Tsr1^b^	CGAAAACATACGACAACTGC	87–250 bp

Degenerate primers for amplification of *COI* were designed for the present study. Primers targeting *ESV* were used for *Trichinella* genotyping in previous studies (^a^Masny *et al*.^20^; ^b^Zarlenga *et al*.^9^). Polymorphic nucleotides are highlighted in bold; those used as an anchor to increase the primer’s melting temperature are indicated by lowercase letters.

The qPCR amplifications of the polymorphic *COI* and *ESV* regions were performed immediately prior to HRMA by LightCycler 480 (Roche). Samples were tested in duplicate in two independent runs. The FW2-uniREV primer pair for the *COI* region and uniTrich1bis and Tsr1 for the *ESV* region were used (Table 2). For both markers, the qPCR was performed in a final volume of 20 µl: 1X Kapa HRM FAST Master Mix (Kapa Biosystems) containing Eva Green saturating dye, 2.5 mM MgCl_2_, 250 nM of each primer, PCR H_2_O (Top-Bio) up to 17 µl, and 3 µl of genomic DNA from a single ML. PCR started with enzyme activation at 95 °C for 3 min followed by 45 cycles of 95 °C for 5 s, 57 °C for 40 s, and a final cooling step of 40 °C for 30 s. HRMA analysis was carried out in a temperature range from 70 °C to 90 °C with data acquisition every 0.02 °C. For the subsequent analysis, the T_m_ Calling and Gene Scanning options of the LightCycler 480 software (version 1.5.0.39) were used.

### PCR and DNA sequence analysis

To reconfirm if the recorded *Trichinella* species-specific matrix curves correspond to predefined reference species (coded isolates), partial *COI* and *ESV* sequences were obtained from all samples included in the HRMA experiment. For this purpose, routine (nonquantitative) PCR was used to amplify both regions. For the *COI* gene amplification, the FW1-uniREV primer pair was used and for the *ESV* region the uniTrich1bis-ESV-Rev1 primer pair was employed (Table 2). PCR was carried out in 8-vial PCR strips in a final volume of 40 µl: 1X FastStart PCR Master (Roche), 500 nM of each primer, ultrapure PCR H_2_O (Top-Bio) up to 37 µl, and 3 µl of genomic DNA from a single ML. Amplification of DNA proceeded as follows: denaturation at 94 °C for 4 min followed by 40 cycles at 94 °C for 10 s, 55 °C for 20 s, 72 °C for 1 min, and a final elongation step of 72 °C for 5 min. The PCR products (35 µl) were purified using the QIAquick PCR Purification Kit (QIAGEN) and eluted into 30 µl H_2_O. Samples were sequenced twice in 20-µl reactions containing 200 ng of purified PCR product and 20 pmol of primer, following the instructions of the Mix2Seq Kit (Eurofins Genomics). DNA sequences were then analyzed and aligned by BioEdit (version 7.2.5). Individual sequences were compared with the sequences in the NCBI database using the Basic Local Alignment Search Tool (BLAST). Sequences, which were identical to sequences previously deposited in the NCBI database were assigned to the appropriate accession numbers (see Supplementary Table S3) and sequences determined as DNA locus-specific for the investigated species were newly deposited under the following accession numbers: for *COI*, *T*. *britovi* (MF402920), *T*. *nelsoni* (MF402921), and *T*. *murrelli* (MF402922); for *ESV*, *T*. *britovi* (MF416213, MF416214), *T*. *pseudospiralis* (MF416215), and *T*. *murrelli* (MF416216).

### Data analysis

The LightCycler 480 version 1.5.0.39 (Roche) enables the analysis of data using T_m_ Calling and Gene Scanning softwares. After the HRMA experiment, the T_m_ Calling software analyzes melting temperatures, course, height, and width of the melting curve peaks of all samples. The Gene Scanning software analyzes data regarding the specific course of sequence melting during HRMA and enables conversion of data by step of normalization, temperature shifting and difference plot formation.

Using Gene Scanning software, the results were normalized by setting the pre-melt (initial fluorescence) and post-melt (final fluorescence) signals of all samples to uniform, relative values from 100% to 0%. In the tests based on the *COI* target gene, values were set to 74.87–75.59 °C and 80.42–81.25 °C; then, the normalized data were shifted (threshold 1) along the temperature axis to equalize the denaturation points of all samples. In the case of the *ESV* target region, melting data were normalized at 72.2–73.89 °C and 91.04–92.03 °C and shifted (threshold 0). To highlight the differences in melting curve shape and to cluster the samples into groups, normalized and shifted data were subtracted from a reference curve to create a difference plot. From each procedure step of the software analysis were extracted raw data and average values for sample duplicates from both runs were calculated. In order to create HRM species-specific matrix curves, these values were used to calculate the median for a particular species. Finally, 95% confidence intervals were also established. The minimum and maximum values of intervals were established by subtraction and addition of margin of error from the median. Margin of error counted with values of standard deviation, two-tailed inverse of the Student’s t-distribution and number of samples.

### Data Availability

All data generated or analyzed during this study are included in this published article (and its Supplementary Information file).

## Results

### qPCR/HRM analysis with reference isolates and blind samples

**Figure 1:**
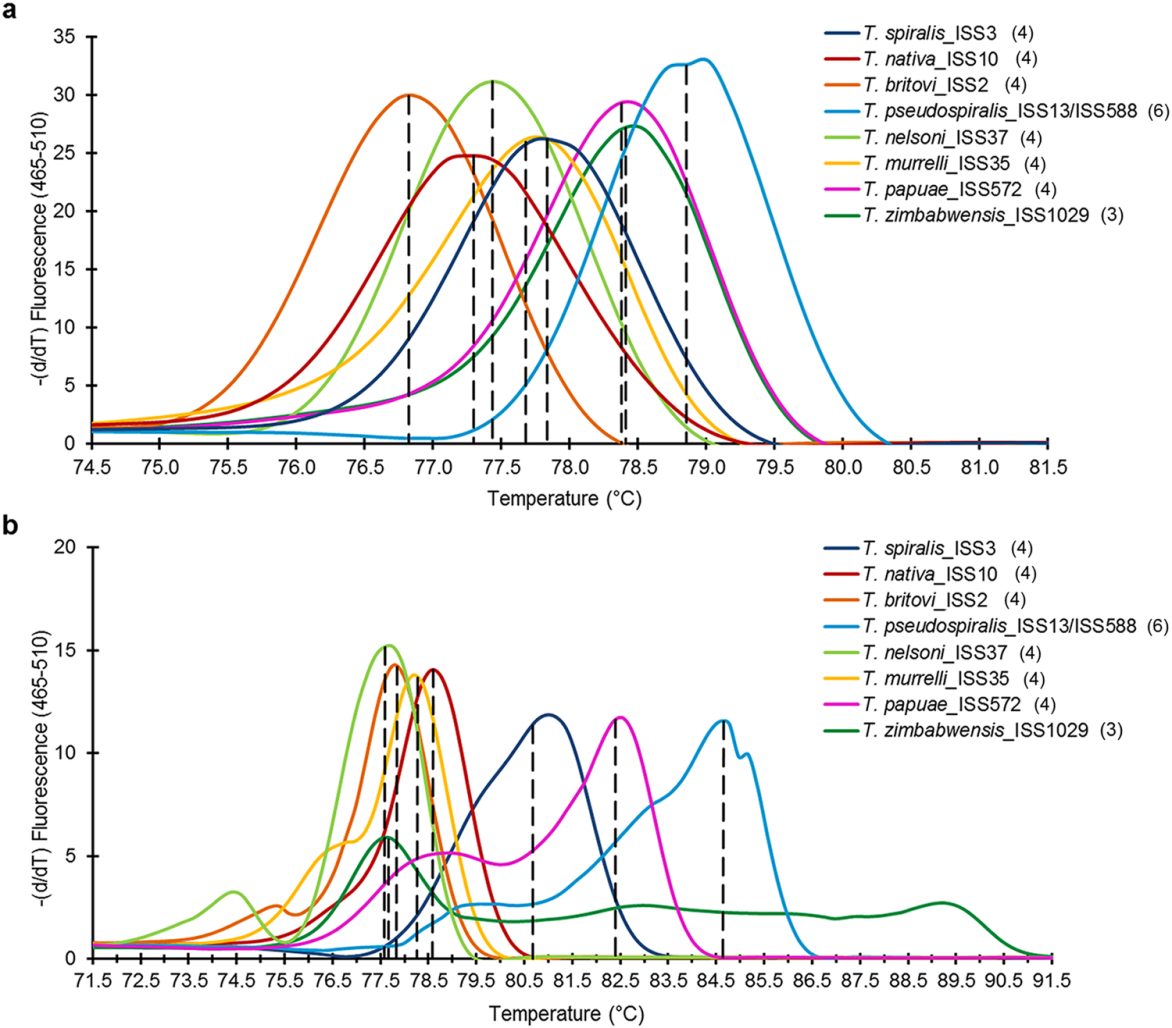
T_m_ Calling Graph, corresponding to melting analysis of amplified isolates from all *Trichinella* reference isolates (33 samples). In parentheses behind sample names are listed the numbers of tested larvae. Interrupted perpendiculars indicate median values of melting temperatures (T_m_) for each species (as recorded in Supplementary Tables S1 and S2). (**a**) Fragments of the *COI* gene (240 bp); (**b**) fragments of the *ESV* region (87–250 bp).

**Figure 2:**
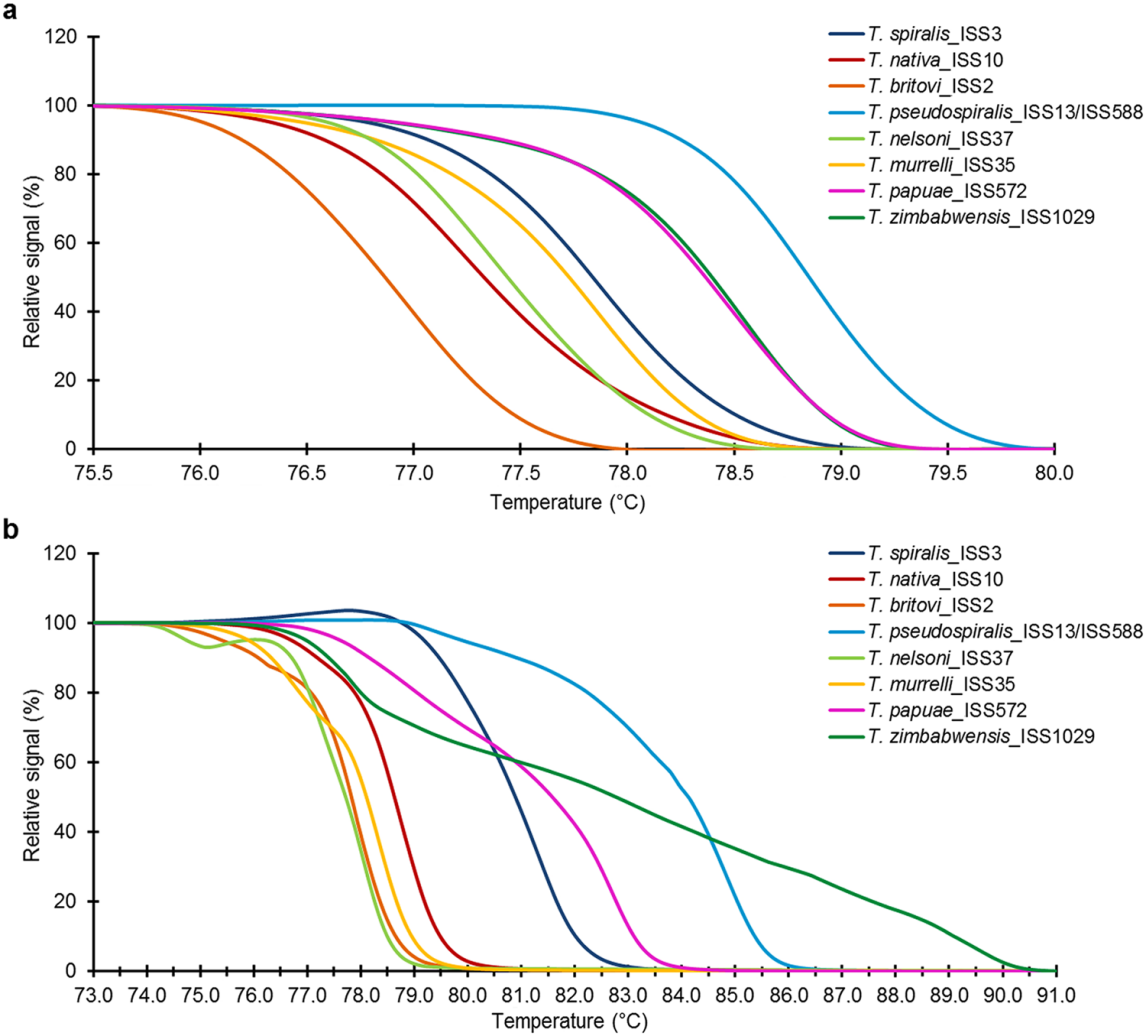
Normalized fluorescence versus temperature resulting from Gene Scanning analysis. (**a**) fragments of the *COI* gene (240 bp) showing that six species could be distinguished; *T*. *papuae* and *T*. *zimbabwensis* could not be distinguished as their curves are overlapping; (**b**) fragments of the *ESV* region (87–250 bp) showed that five species could be distinguished; *T*. *britovi*, *T*. *nelsoni* and *T*. *murrelli* could not be distinguished as the curves were not clearly separated.

**Figure 3:**
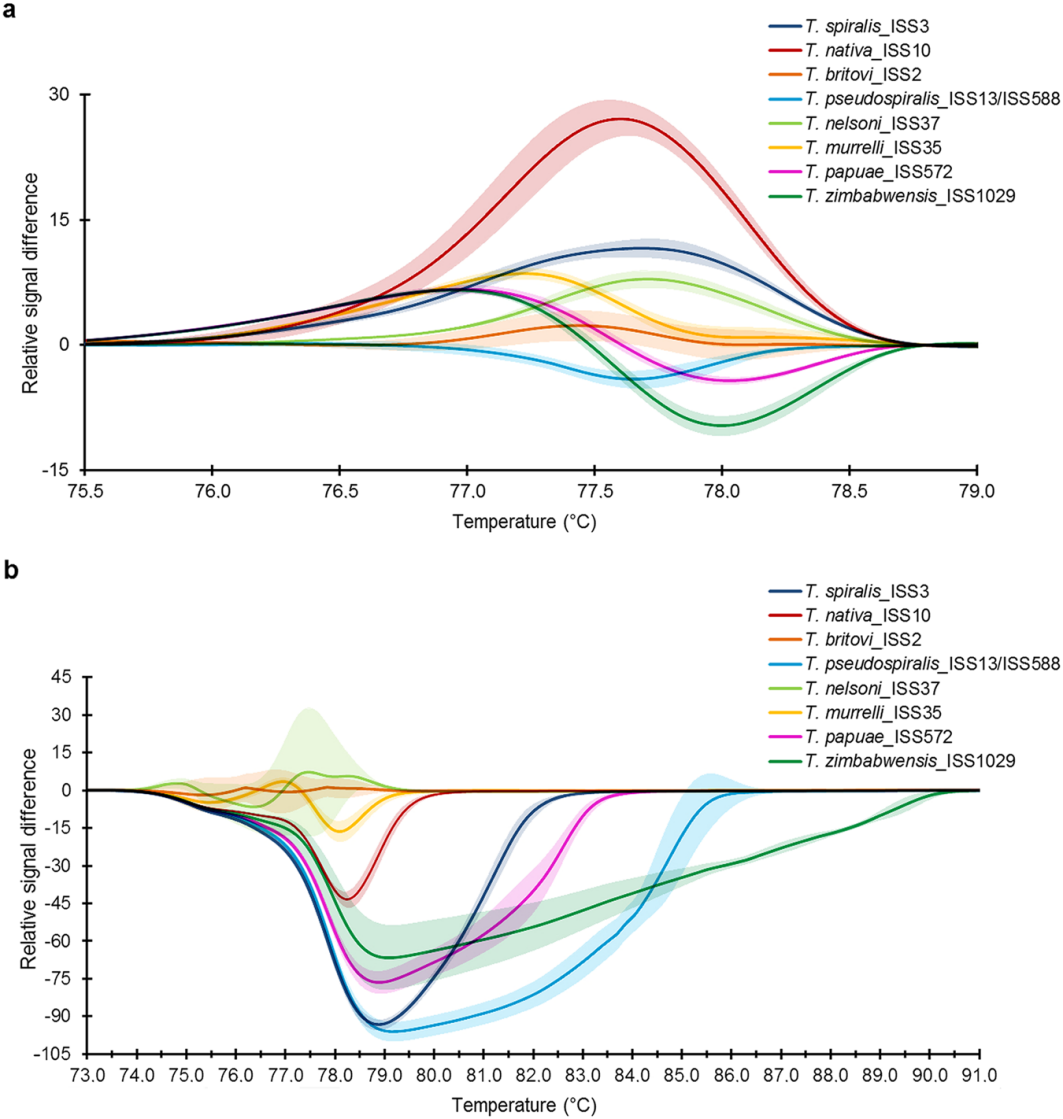
Normalized and Temp-Shifted Difference Plot of HRM species-specific matrix curves with 95% confidence of identification showing reaction progress and sample aggregation. (**a**) *COI* gene fragment PCR amplification with FW2 and uniREV followed by HRMA enabled the distinction of eight *Trichinella* species; (**b**) *ESV* region PCR amplification with uniTrich1bis and Tsr1 followed by HRMA enabling the distinction of five species. *T*. *britovi*, *T*. *nelsoni* and *T*. *murrelli* remained overlapping in the major part of their HRM species-specific matrix curves and confidence intervals, and therefore their reliable differentiation is not possible.

**Figure 4:**
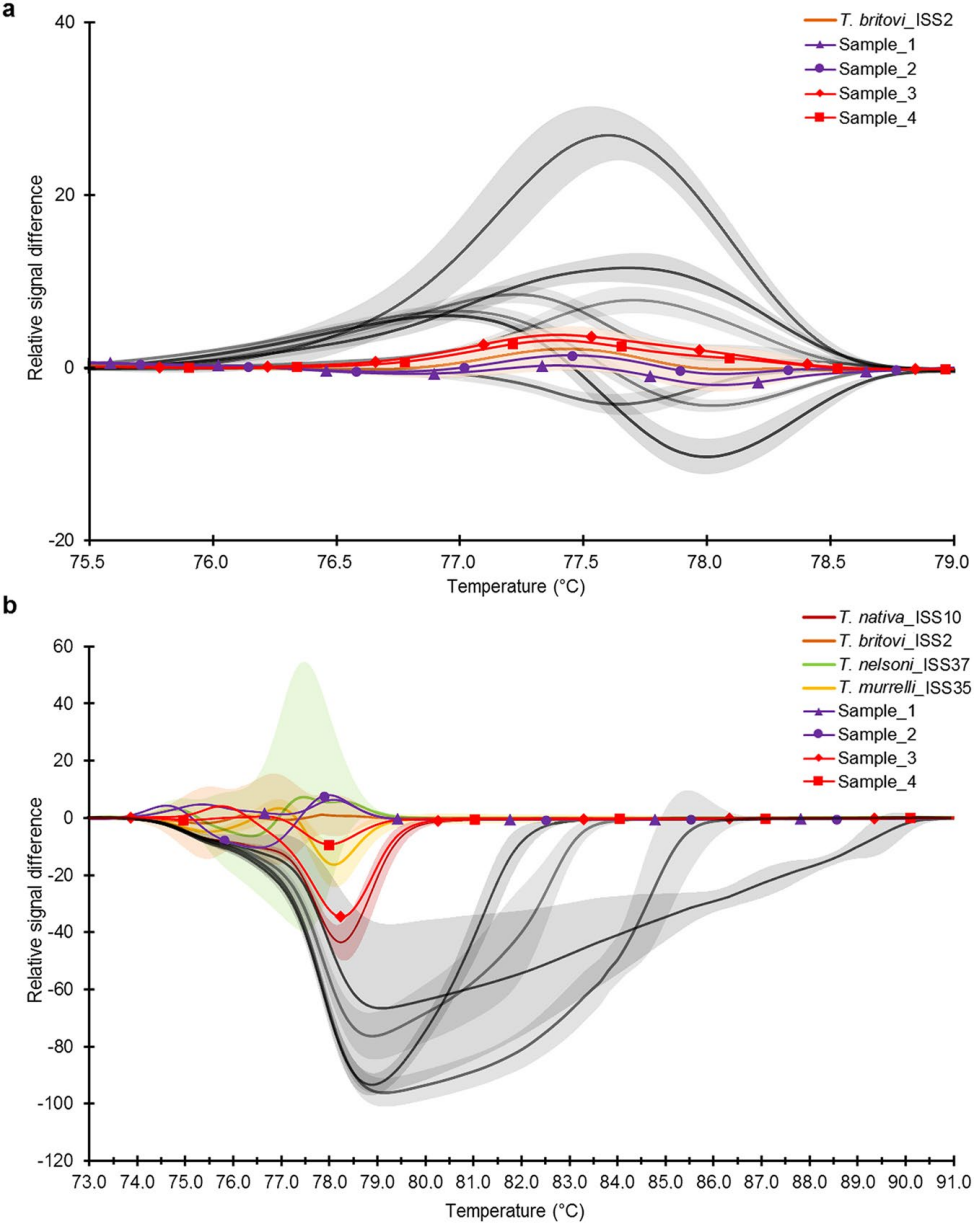
Normalized and Temp-Shifted Difference Plot showing reaction progress in four blind samples from two investigated isolates. (**a**) Based on HRMA of the *COI* gene, blind samples 1–4 clustered in the same group as reference *T*. *britovi* samples at a confidence level of 98%; (**b**) Based on the *ESV* region, samples 1–4 did not cluster clearly in the same group as reference species *T*. *britovi*, and, thus, species identification could not be clearly ascertained even at a confidence level of 99% of identification. Moreover, the HRM species-specific matrix curve data corresponding to sample 3 are closer to reference *T*. *nativa* than *T*. *britovi*.

All larvae tested (33 reference larvae and 4 Polish blind larvae) by FW2-uniREV primer pair of the *COI* gene were amplified resulting in products of 240 bp (same for all investigated species). HRM species-specific melting temperatures (see Supplementary Table S1) and corresponding curve peaks (Fig. 1a) are shown. Six species, except *T*. *papuae* and *T*. *zimbabwensis*, which showed overlapping curves, can be identified from normalized data (Fig. 2a). To present the output data in the most clear and unambiguous manner, a difference plot from the HRM species-specific matrix curves and their 95% confidence intervals were calculated, resulting in eight unambiguously differentiated groups corresponding to the eight species (Fig. 3a). According to HRM species-specific matrix curves, the blind samples (samples 1–4) clustered in the same group as the reference *T*. *britovi* (Fig. 4a), samples 2 and 4 at a 95% confidence level and samples 1 and 3 at a 98% confidence level.

Using the uniTrich1bis-Tsr1primer pair, the *ESV* region fragments for all *Trichinella* larvae (33 samples) and blind samples (sample 1–4) were amplified. Amplicons ranged in length from 87 to 250 bp based on the respective species (*T*. *spiralis*, 134 bp*; T*. *nativa*, 90 bp*; T*. *britovi*, 90 bp*; T*. *pseudospiralis*, 244 and 250 bp*; T*. *nelson*, 116 bp*; T*. *murrelli*, 92 bp*; T papuae*, 199 bp; *T*. *zimbabwensis*, 225 bp; and samples 1–4, 87, 88, and 90 bp). T_m_ Calling analysis of reference isolates enabled generation of HRM species-specific melting temperatures (see Supplementary Table S2) and curve peaks (Fig. 1b). Plotting of statistically processed normalized and transformed data resulted in the clear distinction of five species; whereas curves for *T*. *britovi*, *T*. *nelsoni*, and *T*. *murrelli* were not clearly separated (Fig. 2b). Although the difference plot helped us to distinguish two species that could not be distinguished on the basis of the *COI* target gene, *T*. *britovi*, *T*. *nelsoni*, and *T*. *murrelli* remained overlapping in major parts of their HRM species-specific matrix curves and confidence intervals in the *ESV* region (Fig. 3b). These huge deviations have arisen due to the very different courses of matrix curves and fluctuating melting values of each individual sample. This also affected the determination of the blind samples, in contrast to experiments based on the *COI* gene; HRM species-specific matrix curves based on the *ESV* region did not cluster clearly in the same group as those of the reference species *T*. *britovi* (Fig. 4b), and species identification of these larvae could not be clearly made at any level of confidence. Moreover, the HRM species-specific matrix curve data corresponding to sample 3 were incorrectly closer to reference *T*. *nativa* than to *T*. *britovi*.

### PCR and sequencing

**Figure 5:**
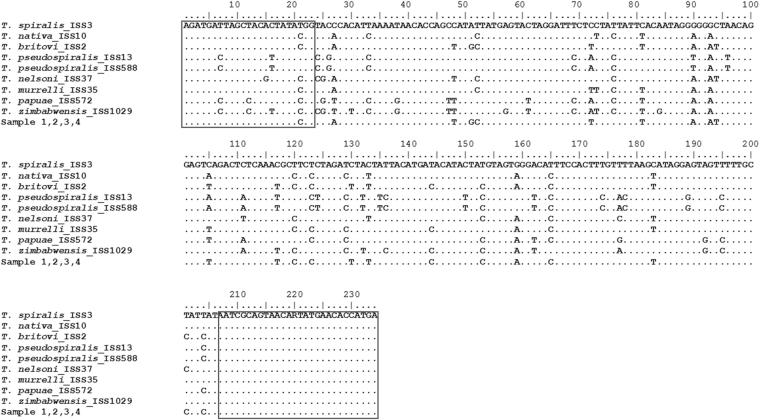
Aligned sequences of the amplified *COI* gene (240 bp) from all 37 *Trichinella* samples (33 reference samples and 4 blind samples). Sequences of all analyzed samples and their duplicates were 100% identical within the respective species. Conserved bases are represented by dots. Variable sites are as indicated. Binding sites of primers FW2 and uniREV are in rectangles.

The *COI* gene DNA fragments of all 37 *Trichinella* samples (33 reference larvae and four larvae of Polish origin as blind control) used in the HRMA were amplified using routine (nonquantitative) PCR with the same primers as above (FW2 and uniREV) and the obtained 531 bp long amplicons were sequenced. Sequencing revealed unexpected SNPs in FW2 primer binding site of *T*. *nelsoni*, *T*. *papuae* and *T*. *zimbabwensis* (Fig. 5); however, these SNPs lied in the middle of the binding site, more than 8 nucleotides far away from the 3′ end and thus did not affect the amplification nor following HRM analysis. Sequences of all analyzed larvae and their duplicates were 100% identical and the relevant sequence differences among the species were confirmed (Fig. 5). Sequences of blind samples 1–4 were 100% identical to each other and also to the reference samples of *T*. *britovi*. These findings correspond to the HRM species-specific matrix curves (Fig. 3a), and confirmed that the DNA of the blind samples belong to *T*. *britovi* (Fig. 4a).

**Figure 6:**
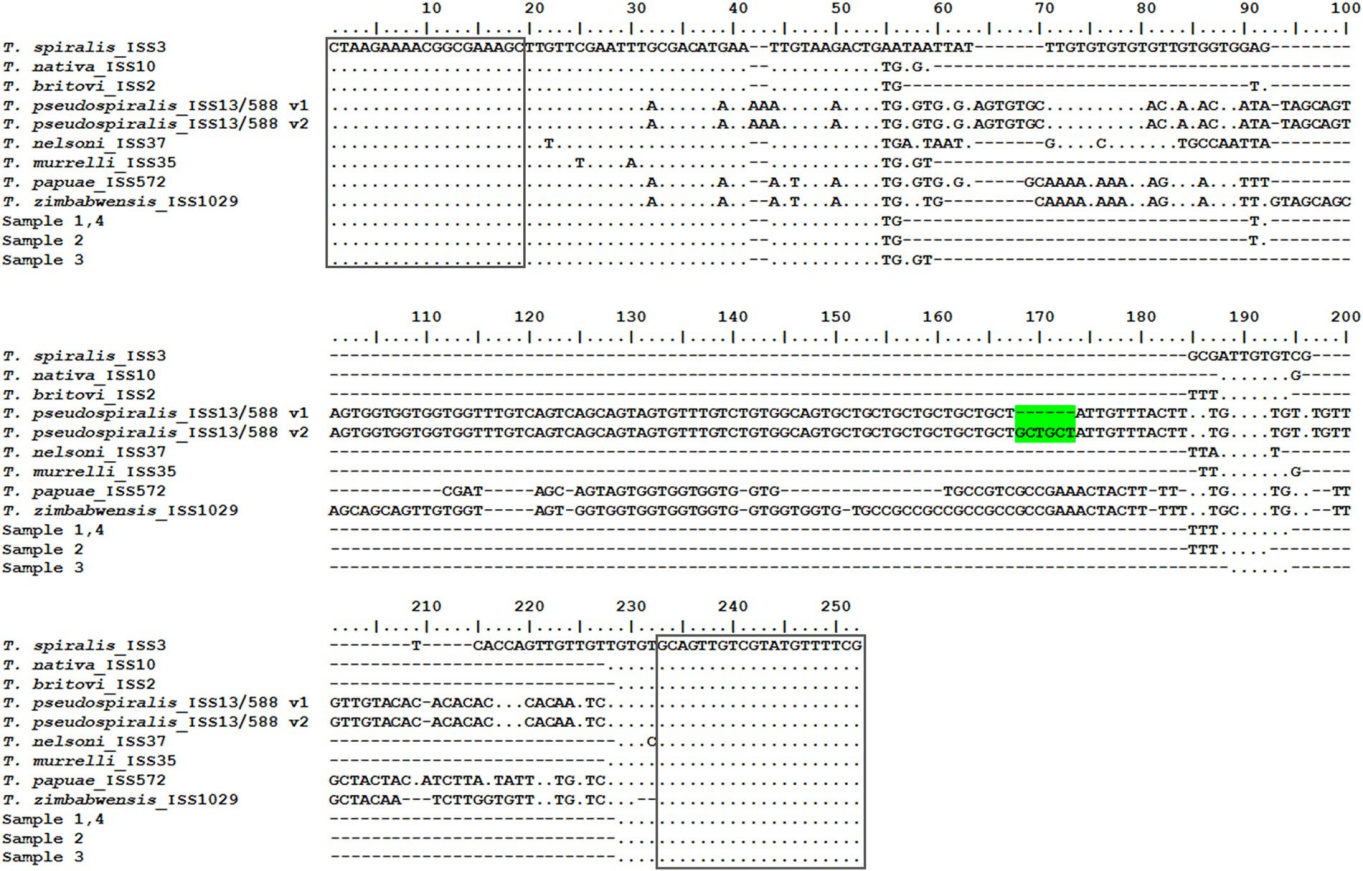
Aligned sequences of the amplified *ESV* region (87–250 bp) of all 37 *Trichinella* samples (33 reference samples and 4 blind samples). Conserved bases are represented by dots, gaps within the sequence by hyphens. Variable sites are as indicated. Binding sites of primers uniTrich1bis and Tsr1 are in rectangles. Polymorphism in the repeat region (two additional GCT repeats) of *T*. *pseudospiralis* was observed in two samples from both isolates and is highlighted in green.

**Figure 7:**
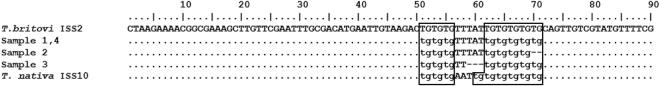
Alignment of the amplified *ESV* region (87–90 bp) of *T*. *britovi* and *T*. *nativa* together with assigned blind samples 1–4. Conserved bases are represented by dots. The repeat regions are indicated by lower case bases and rectangles. TG repeat distribution in *T*. *britovi* ISS2, sample 1 and 4 is the full (TG)_3_TTTAT(TG)_5_; in sample 2 (TG)_3_TTTAT(TG)_4_; in sample 3 (TG)_3_TT(TG)_5_ and in *T*. *nativa* ISS10 (TG)_3_AAT(TG)_6_.

The *ESV* fragments obtained from routine PCR (uniTrich1bis and ESV_Rev1) were sequenced, resulting in products of 313–468 bp (*T*. *spiralis*, 357 bp*; T*. *nativa*, 313 bp*; T*. *britovi*, 313 bp*; T*. *pseudospiralis*, 462 and 468 bp; *T*. *nelsoni*, 345 bp*; T*. *murrelli*, 315 bp; *T papuae*, 420 bp; *T*. *zimbabwensis*, 446 bp; and samples 1–4, 310, 311, and 313 bp). Sequences of samples and duplicates of *T*. *spiralis*, *T*. *nativa*, *T*. *britovi*, *T*. *nelsoni*, *T*. *murrelli*, *T*. *papuae*, and *T*. *zimbabwensis* were 100% identical and the relevant sequence differences among the species were confirmed (Fig. 6). However, two out of six samples of *T*. *pseudospiralis* had an additional GCT_2_ repeat (in green in Fig. 6) resulting in a 6 bp difference in their amplicons. Also, blind samples 2 and 3 showed discrete sequence variants (Fig. 7) in the repeat region (sample 2, (TG)_3_TTTAT(TG)_4_; sample 3, (TG)_3_TT(TG)_5_), while samples 1 and 4 showed identical sequences as (according to the *COI* gene results) the reference *T*. *britovi* ((TG)_3_TTTAT(TG)_5_). These sequence differences caused a great dispersion of HRM species-specific matrix curves of samples 2 and 3 observed in the difference plot (Fig. 4b). The captured sequence variants are, however, not only typical for *T*. *britovi* but also for *T*. *nativa*. In the present study, the four tested *T*. *nativa* larvae carried the conserved repeat variant (TG)_3_AAT(TG)_6_, which probably corresponds to *T*. *britovi*, especially blind sample 3. These observations also correspond with the high similarity of HRM species-specific matrix curves of sample 3 with the matrix curve of *T*. *nativa*, rather than that of *T*. *britovi*. Sequences from both samples 2 and 3 were also aligned using the BLAST tool. The alignment of an 88 bp long fragment of sample 2 revealed 100% homology to *T*. *britovi* ISS392 (JN971026.1), but alignment of an 87 bp long fragment of sample 3 showed only 98% homology to this blasted sequence and 97% homology to *T*. *nativa* ISS10 (JN971020.1), supporting the results of sequencing and the HRM species-specific matrix curves in the difference plot (Fig. 4b).

## Discussion

Until now, *Trichinella* species identification^8–12,14,15^ has been based on multiplex PCR analysis of rDNA fragments (*ESV*, *ITS1*, *ITS2*) and of the variabilities in their lengths, which manifest as a simple and unique electrophoretic DNA banding pattern. Additionally, the gene encoding the 43 kDa excretory/secretory antigen and the *COI* gene are used to differentiate T8 and T9 genotypes from the other species.

The mt *COI* gene holds, due to its sequence conservation, great potential for specific identification and differentiation of numerous species^22^. In the present study, we developed a single-tube qPCR-HRMA method for reliable molecular species determination based on the polymorphism of the *COI* gene and gDNA isolated from a single muscle larva of eight *Trichinella* species. Regarding DNA extraction from single muscle larva, we tested several different approaches (precipitation) and commercial kits (column DNA isolation); no significant variance in efficacy was recorded. However, we can recommend avoiding usage of elution buffers with high salt concentrations and use TE buffer or H_2_O instead. For the extraction of gDNA from single larva (small sample) our extraction protocol proved oneself to be the most suitable considering time consumption, yield and purity of gDNA. *COI* gene fragments of the same length (240 bp) were obtained from all eight test species using the FW2-uniREV primer pair, and all the amplicons subsequently underwent melting analysis. Melting temperatures (T_m_) can act as a guide in species determination; however, by themselves are not always clearly decisive, since some T_m_ peaks are very close to each other or even overlapping. However, after the appropriate transformation of melting curve data a species-specific curve can be generated. Together with the height and width of the melting curve peaks (Fig. 1a), species determination then becomes explicit and reliable. For the evaluation of melting curve data, we used a difference plot enabling the construction of HRM species-specific matrix curves, which could unambiguously differentiate all species (Fig. 3a), with 95% confidence. In order to reconfirm the species-specificity of the recorded qPCR-HRM curves, the corresponding samples were subsequently sequenced and larvae from four blind samples originating from wild boar were tested in the same way. The sequencing analysis confirmed the accuracy of the previously performed qPCR-HRM analysis. Similarly, according to the HRM species-specific matrix curves, amplicons of the *COI* gene of four blind samples were identified as *T*. *britovi*, (Fig. 4a), and confirmed by the sequencing data. There are visible substantial variations between the matrix curves of each blind sample, even though their sequences were 100% identical. The scope of the curves fell into the range of confidence interval; therefore, this is probably caused by the natural noise of HRM analysis. The HRM *Trichinella* species-specific matrix curves (lying with 95% probability within the indicated interval) might potentially find use in a computer application which could, after qPCR-HRMA, automatically compare the shape of an unknown curve to a reference curve and identify by this way the species.

Interestingly, the HRM species-specific matrix curves of all three non-encapsulated species clustered downward on the x-axis resulting in negative values for their relative signal difference (Fig. 3a), in comparison to encapsulated species. This could be caused by the general differences in the mt genomes of *Trichinella* species, which clearly distinguish both groups in the phylogenetic tree^6^.

Recent qPCR-HRMA study by Masny *et al*.^20^ focused on *Trichinella* genotyping based on detection of polymorphisms in LSU rDNA microsatellite sequences of the *ESV* region. Isolate-specific ESV sequence variants (alleles) were used as allelic standards for formation of reference HRM allele-specific matrix curves. These curves were prepared specifically for each single ESV sequence variant or mixtures of sequence variants, imitating allelic composition characteristic for the investigated populations of *Trichinella* isolates. No differences in overlapping matrix curves were observed (from Masny *et al*.^20^ - Fig. 1) between examined pools of larvae from isolates of *T*. *spiralis* (ISS3 and ISS160). However, sequential polymorphisms leading to deviations in HRM allele-specific matrix curves were found in the *T*. *nativa* (ISS10 and ISS70), *T*. *britovi* (ISS2 and ISS392), and *T*. *pseudospiralis* (ISS13 and ISS1348). The results of Masny *et al*. (2012) showed that the genetic diversity between the sequences derived from a single isolate was higher than the inter-isolate variation^20^ of the same parasite species as we demonstrated for *T*. *pseudospiralis*. In the present study, we adopted this existing system and extended it to four other species–*T*. *nelsoni*, *T*. *murrelli*, *T papuae* and *T*. *zimbabwensis*. This dual approach was used to allow better evaluation of the potential of qPCR-HRMA for *Trichinella* species determination.

By the qPCR-HRM analysis of the ESV region, only five (*T*. *spiralis*, *T*. *nativa*, *T*. *pseudospiralis*, *T papuae*, *T*. *zimbabwensis*) out of eight species were clearly differentiated (see difference plot, Fig. 3b). In addition, the HRM species-specific matrix curves of blind samples 2 and 3 (each from a different isolates) did not cluster with *T*. *britovi*, as expected (Fig. 4b), and sequencing revealed polymorphisms in the number of repeats in the microsatellite regions of these larvae (Fig. 7), which contributed to their significant deviation from the reference *T*. *britovi* larvae. Polymorphism in the number of repeats is determined by intra-species and even intra-isolate variability, which is typical for the *ESV* microsatellite region as previously described^9,20,24,25^. BLAST also revealed an uncertain identity of blind sample 3 to *T*. *britovi* ISS392 (98%) and *T*. *nativa* ISS10 (97%); however, no hybrids between these species have been reported, although they are sympatric in some habitats, i.e., Palearctic and Nearctic regions^26^. Nevertheless, there is a high potential of gene flow between sympatric species and genotypes in mixed infections in animals^8^; in case of *T*. *britovi* and *T*. *spiralis* interspecies recombination was confirmed under natural conditions^27^.

Given the nature of microsatellite sequences (such also those of LSU rDNA of the *ESV* region), which are very variable with regard to the number of repeats and/or sequence from individual to individual and which are also mutation-prone, for genotyping studies it would be necessary to investigate a much larger number of isolates and samples and also to use a single larva approach to detect rare alleles. Therefore, the qPCR-HRMA-based strategy focused on the *ESV* region and other non-coding regions is useful for genotyping and screening samples for polymorphisms, but is not reliable for molecular species determination based on melting analysis of single larva. For such purposes, should be probably searched more appropriate target sequence within *ESV*, which would not contain these microsatellite repeats.

## Conclusions

Reliable diagnostics should be followed by appropriate determination of particular *Trichinella* species, which is highly important for a general understanding of the epidemiology of the disease. The results of our qPCR-HRMA study based on mt *COI* gene sequences of PCR products of the same length allow the differentiation of eight *Trichinella* species without the separation of obtained amplicons by DNA electrophoresis and subsequent sequencing; after qPCR-HRM analysis, the *Trichinella* species could be determined on the basis of species-specific matrix melting curves. We envisage that this method could be easily applied in routine diagnostics; after qPCR-HRMA assay, the *Trichinella* species-specific matrix curves could be automatically generated using the computer application and their shape compare to reference curve leading to the identification of particular species.

## Electronic supplementary material

Dataset 1
