# Supplementary material for: Fast and Reliable Differentiation of Eight Trichinella Species Using a High Resolution Melting Assay
Source: Sci Rep. 2017 Nov 24;7:16210. doi: 10.1038/s41598-017-16329-x (PMC5701189; doi:10.1038/s41598-017-16329-x)
Supplement: Supplementary file 1 — Dataset 1 [file 41598_2017_16329_MOESM1_ESM.doc]

**Fast and reliable differentiation of Eight *Trichinella* Species Using a High Resolution Melting Assay**

**Nikol Reslova, Lucie Skorpikova, Michal Slany, Edoardo Pozio, Martin Kasny**

**Table S1: Average values and standard deviations (SD) of melting temperatures (T_m_) of PCR products amplified from the *COI* region.** The values were calculated from each muscle larva of the particular species and their replicates and from all repeats of the performed experiments.

ND – not determined.

| **Species and isolate codes** | **T_m_ (°C**) ± **SD** | | | | | |
| --- | --- | --- | --- | --- | --- | --- |
|  | **T_m_1** | **T_m_2** | **T_m_3** | **T_m_4** | **T_m_5** | **T_m_6** |
| *T. spiralis* ISS3 | 77.97 ± 0.11 | 77.86 ± 0.04 | 77.81 ± 0.05 | 77.69 ± 0.03 | ND | ND |
| *T. nativa* ISS10 | 77.13 ± 0.04 | 77.14 ± 0.03 | 77.55 ± 0.04 | 77.46 ± 0.01 | ND | ND |
| *T. britovi* ISS2 | 76.91 ± 0.01 | 76.88 ± 0 | 76.76 ± 0 | 76.69 ± 0.01 | ND | ND |
| *T. pseudospiralis* ISS13, ISS588 | 79.07 ± 0.04 | 79.00 ± 0.04 | 78.90 ± 0.04 | 78.81 ± 0.06 | 78.75 ± 0.06 | 78.78 ± 0.02 |
| *T. nelsoni* ISS37 | 77.49 ± 0.03 | 77.43 ± 0.09 | 77.44 ± 0.06 | 77.43 ± 0.01 | ND | ND |
| *T. murrelli* ISS35 | 77.73 ± 0.03 | 77.68 ± 0 | 77.68 ± 0.02 | 77.55 ± 0.14 | ND | ND |
| *T. papuae* ISS572 | 78.37 ± 0.02 | 78.4 ± 0.04 | 78.36 ± 0.01 | 78.39 ± 0 | ND | ND |
| *T. zimbabwensis* ISS1029 | 78.48 ± 0.06 | 78.41 ± 0.02 | 78.33 ± 0.01 | ND | ND | ND |
| Sample 1,2 | 76.67 ± 0 | 76.72 ± 0.02 | ND | ND | ND | ND |
| Sample 3,4 | 77.1 ± 0.04 | 77.03 ± 0.01 | ND | ND | ND | ND |

**Table S2: Average values and standard deviations (SD) of melting temperatures (T_m_) of the PCR products amplified from the *ESV*** **region during HRMA.** The values were calculated from each muscle larva of the particular species and their replicates and from all repeats of the performed experiments.

**ND – not determined.**

| **Species and isolate codes** | **T_m_ (°C**) ± **SD** | | | | | |
| --- | --- | --- | --- | --- | --- | --- |
|  | **T_m_1** | **T_m_2** | **T_m_3** | **T_m_4** | **T_m_5** | **T_m_6** |
| *T. spiralis* ISS3 | 80.84 ± 0.09 | 80.70 ± 0.02 | 80.66 ± 0.01 | 80.63 ± 0.03 | ND | ND |
| *T. nativa* ISS10 | 78.52 ± 0.04 | 78.55 ± 0.02 | 78.63 ± 0 | 78.61 ± 0.04 | ND | ND |
| *T. britovi* ISS2 | 77.82 ± 0.02 | 77.83 ± 0 | 77.81 ± 0.01 | 77.94 ± 0.01 | ND | ND |
| *T. pseudospiralis* ISS13, ISS588 | 82.91 ± 1.80 | 84.65 ± 0.09 | 84.65 ± 0.01 | 84.64 ± 0.05 | 85.36 ± 0.02 | 85.35 ± 0.03 |
| *T. nelsoni* ISS37 | 77.19 ± 0.05 | 77.72 ± 0.04 | 77.64 ± 0.04 | 77.53 ± 0.06 | ND | ND |
| *T. murrelli* ISS35 | 78.21 ± 0.02 | 78.29 ± 0.01 | 78.24 ± 0.01 | 78.27 ± 0 | ND | ND |
| *T. papuae* ISS572 | 82.29 ± 0.02 | 82.47 ± 0.01 | 82.31 ± 0.04 | 82.48 ± 0.05 | ND | ND |
| *T. zimbabwensis* ISS1029 | 77.56 ± 0.03 | 77.67 ± 0.12 | 77.70 ± 0.28 | ND | ND | ND |
| Sample1,2 | 76.24 ± 2.14 | 77.59 ± 0.01 | ND | ND | ND | ND |
| Sample3,4 | 74.97 ± 0 | 78.01 ± 0.03 | ND | ND | ND | ND |

**Table S3: Identity of examined sequences to sequences with accession numbers, deposited in the NCBI GenBank database. In bold are highlighted accession number of sequences, which were in this study determined as DNA locus-specific and deposited as new sequences in the database. Sequence lengths inside brackets denote the different variants of the obtained products (see section 3.2 PCR and sequencing).**

| **Species** | **Accession number** | |
| --- | --- | --- |
|  | ***COI*** | ***ESV*** |
| *T. spiralis* ISS3 | AF293969.1 | AF342803.1 |
| *T. nativa* ISS10 | AP017702.1 | JYDW01000638.1 |
| *T. britovi* ISS2 | **MF402920** | **MF416213** (311 bp)  **MF416214** (310 bp)  JYDI01000389.1 (313 bp) |
| *T. pseudospiralis* ISS13, ISS588 | KM357409.1 | **MF416215** (462 bp)  JYDR01000227.1 (468 bp) |
| *T. nelsoni* ISS37 | **MF402921** | JYDL01000264.1 |
| *T. murrelli* ISS35 | **MF402922** | **MF416216** |
| *T. papuae* ISS572 | KM357417.1 | JYDO01000254.1 |
| *T. zimbabwensis* ISS1029 | JYDP01000282.1 | JYDP01000256.1 |
